# LAM2: An Unusual Laminaran Structure for a Novel Plant Elicitor Candidate

**DOI:** 10.3390/biom13101483

**Published:** 2023-10-05

**Authors:** Cathleen Mirande-Ney, Quentin Arnaudin, Gaëlle Durambur, Carole Plasson, Sophie Bernard, Christophe Chamot, Julie Grivotte, Narimane Mati-Baouche, Azeddine Driouich, Jeremy Brebion, Franck Hennequart, Patrice Lerouge, Isabelle Boulogne

**Affiliations:** 1 GLYCOMEV UR 4358, SFR Normandie Végétal FED 4277, Innovation Chimie Carnot, University of Rouen Normandie, IRIB, F-76000 Rouen, France; 2 INSERM, CNRS, HeRacLeS US 51 UAR 2026, PRIMACEN, University of Rouen Normandie, F-76000 Rouen, France; 3 ALGAIA R&D Center, 91 Rue Edouard Branly, F-50000 Saint-Lô, France

**Keywords:** plant defense, structural characterization, succinylated glucan, *Laminaria hyperborea*, gene expression analysis, cell wall immunofluorescence labeling

## Abstract

Laminarans are of interest because they have been shown to induce various immune responses in animals and plants. These β-D-glucans differ from each other by their branching rate, which is possibly responsible for their biological activities. In the present study, we characterized a laminaran fraction extracted from *Laminaria hyperborea* and named LAM2 using sugar composition and structural analyses (NMR). Then, we evaluated its activity as a potential plant elicitor in vitro on tomato seedlings using gene expression analysis and cell wall immunofluorescence labeling. Our study showed that LAM2 isolated from *L. hyperborea* is a succinylated laminaran which significantly enhanced the plant defense of tomato seedlings and induced cell wall modifications, suggesting a higher elicitor activity than the laminaran standard extracted from *Laminaria digitata*.

## 1. Introduction

Laminarans, also called laminarins, are bioactive components that are extracted from brown seaweeds [1] like *Saccharina latissimi* (L.) [2], *Laminaria hyperborea* (Gunnerus) Foslie, *Laminaria digitata* (Hudson) J.V.Lamour., *Ecklonia kurome* Okamura, or *Eisenia bicyclis* (Kjellman) Setchell. They are one of the most abundant carbon sources found in marine ecosystems, representing up to 35% of the dry weight in some seaweeds [1]. Laminarans are of interest because they have been shown to induce antimicrobial and anticancer activities and to enhance the host immune system in animals [3]. In plants, they also induce various defense and resistance reactions as elicitors in rice [4], *arabidopsis*, tobacco [5], tobacco cell suspensions [6], alfalfa [7] and grapevine [8]. In these cases, the defense responses were found to activate Pattern-Triggered Immunity (PTI) including plant hormone signaling, the phenylpropanoid pathway and cell wall modifications [6,9,10].

Laminarans are generally low-molecular-weight (e.g., 5 kDa) β-D-glucans which consist of β-(1 → 3)-linked D-glucose residues. They differ from each other regarding their lengths and branching structures. Indeed, they may contain some 6-O-branches in their backbones and some β-(1 → 6)-intrachain links [6,9,10].

Laminarans are present in either soluble or insoluble forms. Their solubility is influenced by the degree of branching. In fact, highly branched laminarans are soluble in cold water whereas lower levels of ramification induce solubility only in warm water [11]. Two types of laminarans have been described: one type with chains that are terminated by D-mannitol residues (M-series) and another type with chains terminated by D-glucose residues (G-series) [12]. Interestingly, several laminarans have been shown to have structures quite distinct from that originally suggested for the commercially available preparations from *Laminaria* sp. [13]. That could be explained by the fact that the ratios of the two types of laminarans, as well as their structures, can vary according to the seaweed species, as well as environmental factors such as nutritive salts, temperature or frond age [13,14]. For example, laminarans extracted from the brown seaweed *Saccharina longicruris* (Bachelot Pylaie) Kuntze at different harvesting periods showed structural modifications of their O-6 branching rate and of their conformational structure [15]. These factors are also believed to influence the biological activity of laminarans.

In the present study, we characterized a laminaran fraction extracted from *Laminaria hyperborea* and named LAM2 using sugar composition and nuclear magnetic resonance (NMR) analyses and compared it to laminaran from *Laminaria digitata*. Then, we have evaluated its activity as a potential plant elicitor in vitro on tomato seedlings using gene expression analysis and cell wall immunofluorescence labeling.

## 2. Materials and Methods

### 2.1. Plant Material and Treatment

*Solanum lycopersicum* L. seeds (cv. St Pierre) were sterilized with ethanol and sodium hypochlorite and cultivated on agar ½ MS medium at 24 °C (80–90% relative humidity (RH), 16 h to 8 h day and night cycle), in a growth chamber for 13 days. The seedlings were elicited with water, 1% (*w*/*v*) standard laminaran from *L. digitata* (LAM std) or 1% (*w*/*v*) laminaran LAM2 (from *L. hyperborea*) three days after sprouting. Root and aerial parts lengths were measured using the ImageJ software (1.54f version).

The standard laminaran (LAM std) was obtained from Sigma-Aldrich (L9634; Saint-Quentin-Fallavier, France). The laminaran LAM2 fraction was isolated using an industrial targeted *Laminaria hyperborea* extraction process involving ethanol precipitation. Precipitated laminaran was resuspended in water and recovered from fucoidans using an ultrafiltration step.

### 2.2. Structural Identification

The sugar composition of LAM2 was determined using anion exchange chromatography after acid hydrolysis. Briefly, 20 mg of LAM2 were hydrolyzed in 1 mL of a 4 M TFA solution at 100 °C for 4 h. The hydrolysate was then injected to a thermoElectron ICS5000+ system and monosaccharides were separated on a CarboPac SA10 column, eluted with 1 mM KOH, and detected with pulsed amperometric detection.

For nuclear magnetic resonance (NMR), ^1^H and ^13^C NMR spectra were recorded in 100% D_2_O on a 400 MHz Bruker AVIIIHD NMR equipment (Bruker, Wissembourg, France) resonating at 400.25 MHz for ^1^H and operating at 298 K. The solvent resonance was used as the internal standard. Chemical shifts (δ) were quoted in parts per million (ppm). For saponification of LAM2, 10 mg of LAM2 were incubated for 18 h at 20 °C in 0.1 M NaOH, neutralized, dialyzed against water and finally lyophilized. For quantification of succinate, LAM2 and LAM std were hydrolyzed by 2 M TFA for 2 h at 100 °C. Then, presence of succinate was determined in acidic hydrolysates using a succinate colorimetric assay kit (MAK184, Sigma-Aldrich, St. Louis, MO, USA) according to the manufacturer’s recommendations.

### 2.3. Gene Expression Analysis

Total RNAs were extracted from 13-day-old seedlings grown under control and laminaran-spray conditions (LAM2 or LAM std) using the Macherey Nagel NucleoSpin RNA Plant kit. The reverse transcription was performed from 1 μg of total RNAs using the Applied Biosystems High Capacity cDNA Reverse Transcription kit with RNase inhibitor. The reaction mix was prepared in a 96-well reaction plate using the Fast SYBR Green Master Mix (Applied Biosystems, Waltham, MA, USA) in a final volume of 13 μL with 200 nM of each primer and 3 μL of cDNA template. The selected genes *LOXD* (forward primer 5′ CGGAGAGTCGTGTCGAGA 3′, reverse primer 5′ TTAAGCCTGGAGGTTGAGAATG 3′, amplicon size 97 bp), *PAL-5* (forward primer 5′CGGTGTGACTACTGGATTTGG 3′, reverse primer 5′ CTGCCCTTGTTGCTGAATGT 3′, amplicon size 152 bp), *PPO-D* (forward primer 5′ GGCTTAGGAGGTCTTTATGGTG 3′, reverse primer 5′ ATCAGGAGGTGGTGTAGGAG 3′, amplicon size 75 bp), *Pti-5* (forward primer 5′ ATTCGCGATTCGGCTAGACATGGT 3′, reverse primer 5′ AGTAGTGCCTTAGCACCTCGCATT 3′, amplicon size 119 bp), *Worky28* (forward primer 5′ ACAGATGCAGCTACCTCATCCTCA 3′, reverse primer 5′ GTGCTCAAAGCCTCATGGTTCTTG 3′, amplicon size 100 bp), *Worky70-80* (forward primer 5′ GGGCCAGATCGAGGAAGTTG 3′, reverse primer 5′ GCCCATATTTTCTCCATGCACA 3′, amplicon size 167 bp), were amplified using real-time PCR. Two other genes were used as reference *Ef1* (forward primer 5′ GGAACTTGAGAAGGAGCCTAAG 3′, reverse primer 5′ CAACACCGACAGCAACAGTCT 3′, amplicon size 158 bp) and *PHD* (forward primer 5′ ATTCGTGGCTGCTCTCTGTC 3′, reverse primer 5′ CCCTGTCACGGCTTCAAAGA 3′, amplicon size 116 bp). The quantitative real-time PCR was performed using the CFX96 real-time system (Bio-Rad, Hercules, CA, USA). The following parameters were used: 20 s at 95 °C then 40 cycles of 5 s at 95 °C, 20 s at 60 °C followed by the melt-curve analysis: 15 s at 95 °C, 6 min at 60 °C and 15 s at 95 °C. Data were analyzed using the CFX Maestro software (Bio-Rad, Hercules, CA, USA). The relative expression was normalized with the 2^−∆∆Cq^ method to Ef1 and PHD as reference genes using GENorm analysis (provided in CFX Maestro software 2.3 version).

### 2.4. Resin Embedding and Immunofluorescence Labeling

We collected 0.5 cm long sections of the stem, just under the hypocotyl on the 13-day-old seedlings treated with water, 1% LAM Std or 1% LAM2. Sections made on three seedlings for each condition were processed at 4 °C with the electron microscopy tissue processor (EM-TP, Leica Microsystems, Wetzlar, Germany) as follows. Samples were fixed for 1 h 30 mins in 1% paraformaldehyde and 1% glutaraldehyde mixture (*v*/*v*) in 0.1 M sodium cacodylate buffer (pH 7.2) and washed (4 × 5 min) in ultrapure water. Then, the samples were dehydrated in an ethanol series (30%, 50%, 70% and 2 × 100%) for 2 h each and embedded in LRW resin through a LRW—ethanol series (25%, 50%, 75% and 100%), for 24 h each. Finally, they were embedded (6 × 24 h) in LRW resin complemented with the UV catalyst benzoin methyl ether (0.5% *w*/*v*) and polymerized for 48 h at 4 °C with UV light. Sections from resin blocks (2 µm; EM UC6 Leica microsystems) were collected on 10 well slides previously coated with poly-L-lysine (0.01% *v*/*v*).

The resin sections were blocked in PBS-Tween 20 0.1% (*w*/*v*) supplemented with 3% of BSA (bovin serum albumin) and NGS 1/20 (normal goat serum, *v*/*v*) for 30 min. Sections were washed in PBS-T + 1% BSA (5 × 5 min) and incubated overnight at 4 °C in a wet chamber with the primary antibody (see list in Supplementary Materials Table S1). After washing in PBS-T + 1% BSA (5 × 5 min), the sections were incubated for 2 h at 25 °C in a wet chamber with a rat or mouse secondary antibody coupled to Alexa 488 (d:1/200, In Vitrogen). Finally, they were washed in PBS-T + 1% BSA (5 × 5 min) and mQ water (2 × 5 min). Fluorescence on sections was observed using a macroscope Axiozoom Zeiss with a fluorescence filter set 38 HE (BP 470/40 BP 525/50), an exposure time of 400 ms and a range of 50 µm, slides: 11, interval: 5 µm. Negative controls were performed by the omission of the primary antibody.

### 2.5. Image Analysis and Statistical Test

Fluorescence intensity measurements were performed on a region of interest (ROI) containing all the tissues of the stem and used to obtain a mean of fluorescence intensity value of the ROI. To automate the measurements, an ImageJ macro was developed. It manages the opening of an image, the projection of average intensity along the Z axis and the retrieval and saving of intensities along a line in a result table. Z-average projection is used to smooth out cutting effects, as each point on the line is itself an average of a user-defined orthogonal segment. The macro code is provided in Supplementary Materials.

Statistical analysis was performed using a Kruskal–Wallis test followed by multiple pairwise comparisons using Dunn’s procedure at the significance level 0.05 with the statistical software XLSTAT (2018.1.1 software, Copyright Addinsoft 1995–2020).

## 3. Results

### 3.1. Structural Identification of LAM2 Isolated from L. hyperborea

**Figure 1:**
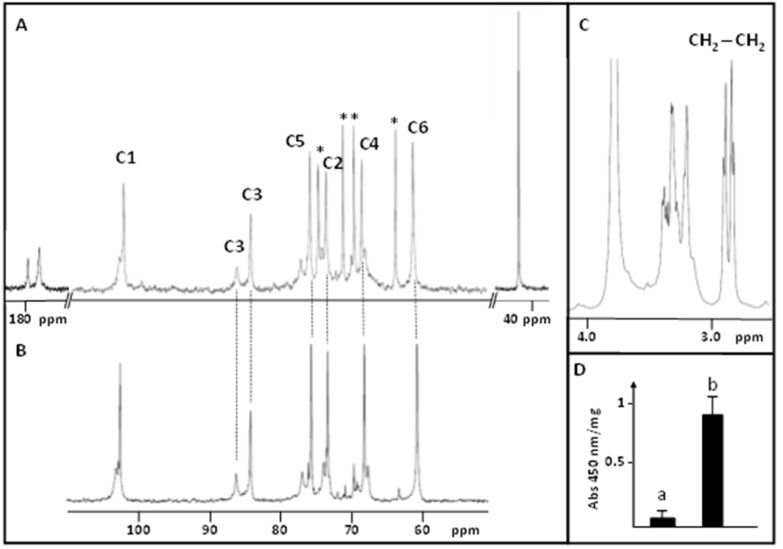
^13^C NMR spectra of (**A**) LAM2 and (**B**) the standard laminaran. * ^13^C signals assigned to the succinylated glucose residues. (**C**) ^1^H NMR spectrum of LAM2. (**D**) Relative quantification of succinate in the standard laminaran (a) and LAM2 (b).

A laminaran fraction was isolated from *L. hyperborea* and named LAM2. Its sugar composition was determined using anion exchange chromatography and indicated that LAM2 is composed mainly of Glc (80%) together with traces of Fuc (7%), Gal (4%), Xyl (5%) and Man (2%). The structure of LAM2 was then investigated using ^1^H and ^13^C NMR spectroscopy, and chemical shifts were compared to a standard laminaran. As illustrated in Figure 1A,B, LAM2 and the standard laminaran exhibited common ^13^C NMR signals that were assigned to C1 to C6 of the β(1,3)-D-glucan backbone and of β(1,6)-branched glucose unit [6,9,10] (Table 1). However, additional signals were also detected in the ^13^C NMR spectrum of LAM2 for C2 to C6 of the glucopyranose residue. This suggested that some Glc units of LAM2 were substituted on C2, C4 or C6, thus resulting in the duplication of ^13^C signals between the substituted and non-substituted glucan backbone. In addition to these ^13^C signals, two signals at δ = 175.69 and 179.46 ppm were assigned to two carboxyl groups together with a methylene group at 43.86 ppm (Figure 1A, Table 1). These data suggested the presence of succinate substituents on LAM2. The detection of two triplets at δ = 2.64 and 2.67 ppm in the ^1^H NMR, that were assigned to the methylene protons of the succinic acid substituent, confirmed the succinylation of the glucan backbone in LAM2 (Figure 1C).

To confirm that LAM2 is a succinylated laminaran, a saponification was performed using 0.1 M NaOH. As expected, after deesterification, the ^13^C NMR spectrum of LAM2 was identical to the one of the standard laminaran. To further confirm the substitution of LAM2 by succinic acid motifs, LAM2 was hydrolyzed using TFA 2 M and the presence of succinate in the hydrolysate was then detected using a succinate colorimetric assay kit by comparison with the standard laminaran (Figure 1D).

**Figure 2:**
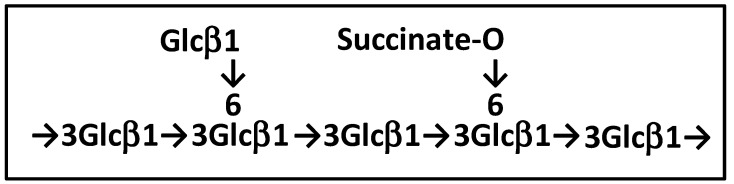
Proposed structure for LAM2.

Considering that the esterification with succinate mainly induced a downfield shift on the C6 ^13^C signal (Δδ of 2.51 ppm), we propose that the succinylation of the laminaran from *L. hyperborea* occurs at C6 of Glc, as depicted in Figure 2. However, we cannot rule out that the succinylation of LAM2 may occur on more than one Glc residue in the repeating motif of LAM2 because the ratio between succinate and Glc was estimated from the integration of ^1^H NMR signals to be about 25 to 33%.

### 3.2. Immunity Gene Expression Analysis

**Figure 3:**
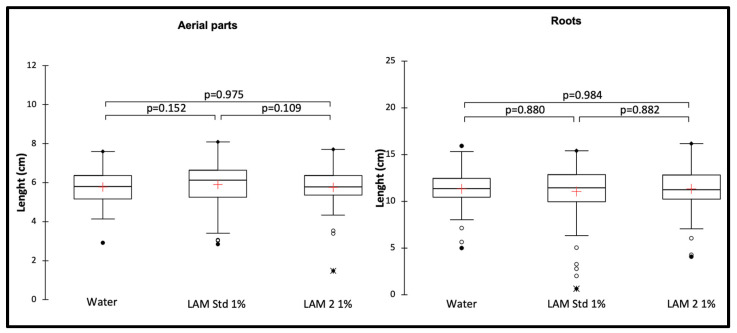
Root and aerial part lengths after treatments with water, LAM2 1% and LAM std 1%. *p*-value according to Kruskal–Wallis test with Dunn’s procedure at the significance level 0.05. The red crosses correspond to the means. The up and down sides of the box are the lower and upper quartiles. The marks (*, ●, ○) are extreme points.

After application of LAM2, LAM std or water, root and aerial part lengths were measured using the ImageJ software. As illustrated in Figure 3, no treatment affected the growth of tomato seedlings.

As laminarans are known to induce defense reactions in various plants [4,5,6,7,8], we investigated the gene expression of six key genes in plant defense: LOXD (a lipoxygenase D in the jasmonic acid pathway), PAL5 (Phenylalanine ammonia-lyase in the phenylpropanoid pathway), PPO-D (a polyphenol oxidase D in the phenylpropanoid pathway), Pti-5 (a pattern-triggered immunity marker), Worky28 and Worky70-80 (Pti marker and transcription factor defense response—salicylic acid related, respectively).

For both laminarans, Worky28 and 70-80 expression were found to be significantly decreased (by −1.68 and −2.28 fold for LAM2; by −2.32 and −1.71 fold for LAM std), respectively as compared to control (water) (Table 2). However, LAM2 seemed to specifically enhance Pti-5 expression by 1.56 as compared to water and PPO-D expression by 1.50 as compared to LAM std. The two other genes (LOX-D and PAL5) seemed to be not significantly expressed as compared to the control (water).

### 3.3. Immunocytochemistry

#### Characterization of Cell Wall Glycopolymers

Cell wall polysaccharides (pectins and hemicelluloses) and hydroxyproline rich glycoproteins (arabinogalactan proteins) were investigated using specific monoclonal antibodies (mAbs, see Table S1 in Supplemental Data) on resin-embedded sections from the stem of the 13-day-old tomato seedlings treated with water, LAM2 or LAM std.

**Figure 4:**
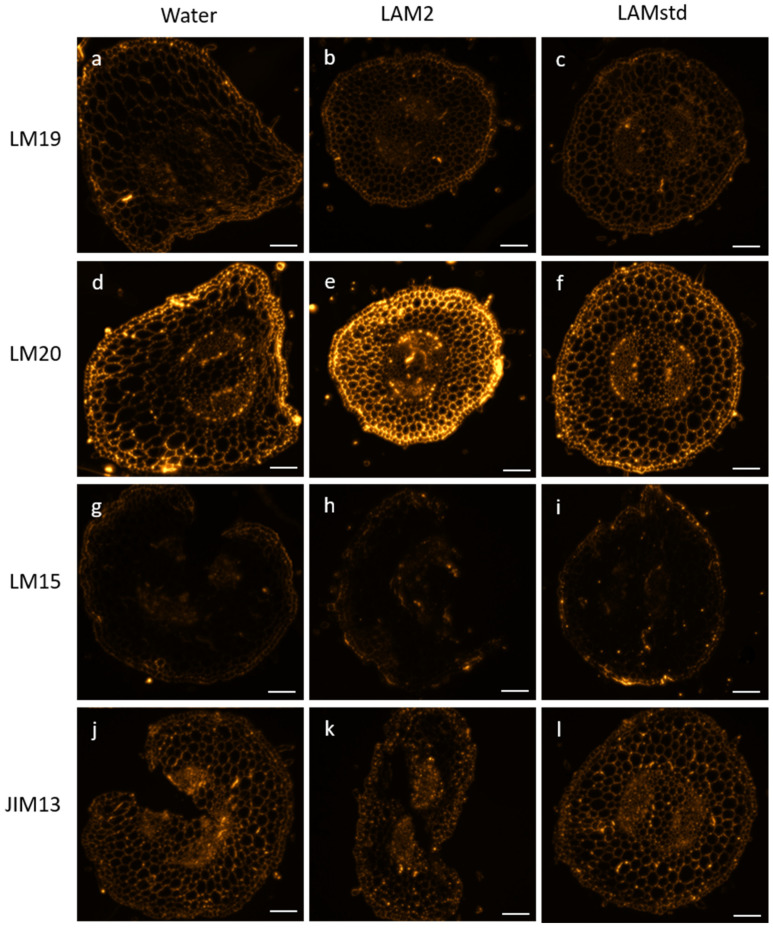
Immunocytochemistry of aerial parts of tomato seedlings on cell wall glycopolymers after treatments with water, LAM2 1% and LAM std 1%. LM19 and LM20 detect weakly or highly esterified homogalacturonans, respectively, LM15 recognize xylosylated xyloglucan epitopes and JIM13 is specific of βGlcA-(1,3)-αGalA-(1,2)-Rha epitope from arabinogalactan proteins. Scale bar: 100 µm.

For each antibody, the fluorescence signals were observed, as summarized in Figure 4. Weakly esterified homogalacturonans (HG) (detected with LM19) showed a weak fluorescence signal in the three conditions (Figure 4a–c), as well as xylosylated xyloglucan recognized by LM15 (Figure 4g–i). Highly esterified HG (LM20) seemed to exhibit a stronger labeling for LAM2 treatment compared to water and LAM std (Figure 4d–f). JIM13 antibody, which recognized βGlcA-(1,3)-αGalA-(1,2)-Rha epitope of arabinogalactan proteins, was detected in the three conditions with a fluorescence signal for LAM2 treatment, which appear to be weaker (Figure 4j–l).

**Figure 5:**
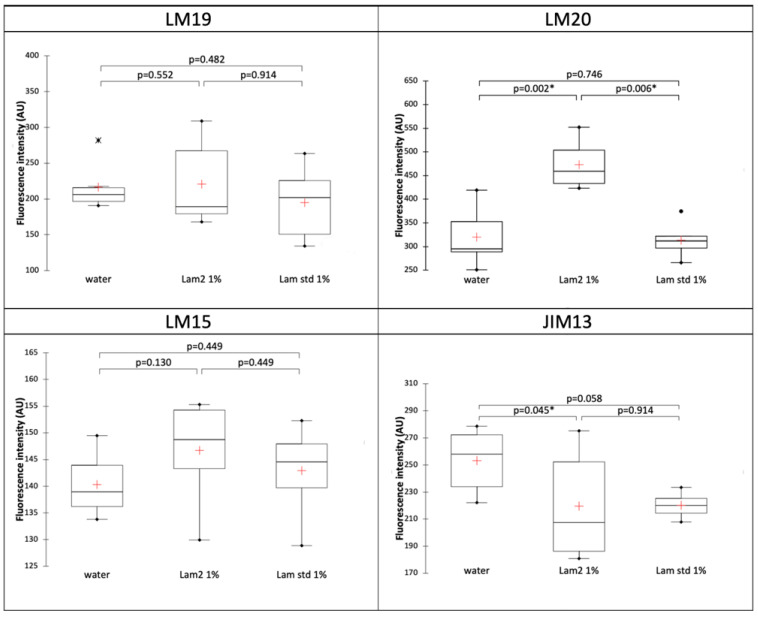
Comparison of fluorescence intensity of cell wall glycopolymer immunostaining after treatments with water, LAM2 1% and LAM std 1%. P-value according to Kruskal–Wallis test with Dunn’s procedure. *: significant at level alpha = 0.05. The red crosses correspond to the means. The up and down sides of the box are the lower and upper quartiles. The marks (*, ●) are extreme points.

To confirm these observations, we then quantified and compared the fluorescence intensity of each antibody between the three conditions (Figure 5). Statistical results corroborate that there is no difference between the three conditions for weakly esterified HG (LM19) and xylosylated xyloglucan (LM15). Also, the results showed a significant increase in highly esterified HG (LM20) for the LAM2 treatment when compared to water or LAM std. Arabinogalactan proteins epitopes (JIM13) presented a significant decrease in the signal for LAM2 treatment compared to water.

## 4. Discussion

Regarding structural identification, our study showed that LAM2 isolated from *L. hyperborea* is a succinylated laminaran. It seems that no “natural” succinylated laminaran has been reported in the literature to date. Succinoglucans are mainly known in bacteria where glucans are substituted with various nonglycosidic residues. In *Brucella abortus* (Hughes) Meyer & Shaw, this substitution is implicated in survival of the bacteria under hypoosmotic conditions where the amounts of succinylated cellular glucans increased with low osmotic pressure of the growth medium [16]. Moreover, as mentioned before, laminarin extracted from other brown seaweeds showed structural modifications on their O-6 branching rate and on their conformational structure in different environmental conditions such as the modification of nutritive salts [15]. It is reasonable to suggest that this original structure could possibly be due to the low osmotic growth conditions of *L. hyperborea* before harvesting and the extraction of LAM2. In addition, although *L. digitata* and *L. hyperborea* are both kelps, they have different natural environments. *L. hyperborea* is a cold-temperate species widely distributed from Portugal to the Norway–Russia border [17]. *L. digitata* occurs in Arctic and cold-temperate regions distributed from Norway to southern Brittany in France and from Greenland to Long Island Sound in USA [18]. *L. digitata* is a perennial species, growing in the infralittoral fringe and upper sublittoral on rocky substrates between 0 and 10 m [18], while *L. hyperborea* dominates the subtidal, shallow, and rocky seabed generally at depths between 10 and 20 m [17]. Moreover, depending on the growth zone, the life cycle of *L. digitata* is shorter (around one year), compared to *L. hyperborea,* which requires 3 to 5 years [17,18]. These different environmental factors could also explain this structural modification between the two kelps.

Our gene expression analysis confirms that both laminarans have plant elicitor activities. Indeed, the Pti markers and transcription factors of defense response (Worky28 and 70-80) had decreased expressions in our conditions. These results are consistent with the literature because in tomato, Workys are studied for their roles in plant defense by either overexpression and/or underexpression. In biotic stresses, several of them, including Worky80, act as negative regulators to enhance the resistance to certain pathogens [19].

Our study showed that LAM2 significatively enhanced the pattern-triggered immunity (with Pti-5 and PPO-D) compared to LAM standard. These results are consistent with previous studies, in which mixed-linked glucans or laminaran up-regulated tomato PTI-marker genes PTI5 and PPO [20,21]. Moreover, the biological activity of oligosaccharides such as β-1,3-glucans is highly dependent on the degree of branching. Plus, some studies have mentioned that plants may have developed the ability to react to structurally different β-glucans [22,23,24]. Thus, it is consistent to think that the succinyl-branching on glucan backbone can possibly enhance LAM2 elicitor activity. However, we cannot exclude at this stage the possible presence of sulfate groups, which might lead to the observed biological activities. Indeed, sulfated laminarans are known to have immune biological activities in animals and plants [25]. These kinds of laminarans have been found in brown seaweeds such as *Fucus* or *Sargassum* genus. Even though no study has reported native sulfated laminarans in *L. digitata* or *L. hyperborea*, the possibility of such cannot be entirely ruled out.

In addition, we detected that our gene expression levels were rather low (1.5-to-2.32-fold change) compared to other studies. This fact can be explained because most of defense gene markers increased with time and exhibited the highest level at 24 h and then rapidly declined and returned to a normal level at 48 h [19]. Yet in our experimental design, gene expression analysis occurred ten days after laminaran application. This clearly indicates that the performance of both laminarans is unaltered for a long time and particularly LAM2 whose effects are higher.

It is known that the first set of defense responses in plants is pathogen-associated molecular pattern-triggered immunity (PTI). Then, plants activate a second immune signaling known as effector triggered immunity (ETI). ETI is stronger and more prolonged than PTI. In addition, PTI and ETI generate immune signals, which in turn activate systemic acquired resistance (SAR). SAR is a form of induced resistance activated after plant elicitor application. PTI, ETI and SAR share several signaling components and immune responses such as activation of anti-microbial metabolites, antioxidant enzymes or phenylpropanoids pathways [26]. Generally, studies in plant immune responses focused on short time scales (minutes or hours), e.g., rather in PTI/ETI scales. Few others dealt with longer time scales (days). One of them evaluates the application of different SAR elicitors 7 and 14 days after treatment. They observed an activation of the levels of defense related proteins including polyphenol-oxidase (PPO) [27]. Thus, regarding to the time scale of plant defense responses, LAM2 could be also SAR elicitor.

Regarding the effects of LAM2 on cell wall glycopolymers, aerial parts of tomato seedlings treated with LAM2 presented an increase in highly esterified HG. To adapt to biotic stress conditions, plant pectin methyl esterase (PME) activity and the level of pectin methylesterification are highly regulated by pathogens during an infection process [28]. Highly esterified HGs are less susceptible to the action of the enzymes produced by the pathogens, which need to degrade the wall components to penetrate and colonize the host tissues [29]. Therefore, highly esterified HG contents are positively correlated with improvement in the resistance against pathogens [29,30,31]. A study on tomato, showed that there is HG with a higher degree of methyl-esterification in resistant genotypes compared to susceptible ones [32].

Contrary to homogalacturonans, we observed a decrease in the arabinogalactan epitope recognized by JIM13. AGPs are involved in several biological processes including functions in response to biotic and abiotic stresses. Generally, studies report an increase in these polymers in the cell wall of plant known to be resistant to pathogens compared to the susceptible plants [33]. However, some others indicated that pathogen inoculation can induce an increase in AGP in susceptible genotypes of tomato [32]. Thus, we can suppose that LAM2 induced an increase in resistance and consequently a decrease in AGP content in tomato seedlings. Moreover, because AGPs contribute to cell wall strengthening via their association with pectin by basic amino acid residues or by calcium-mediated binding, we cannot exclude that the strong labeling of highly esterified HG epitopes masked those of arabinogalactan, preventing them there from being detected.

## 5. Conclusions

Environmental pollution is a worldwide ecological challenge due to, among others, the extensive use of synthetic chemical pesticides. These substances are widely criticized and there is a crucial issue in sustainably enhancing plant resistance to diseases and pests. In this context, plant elicitors are considered beneficial methods to protect plants without affecting environments. They are also tools to better understand their immune system, to engineer plants in identifying the pathways that activate plant defense.

Our study showed that LAM2 isolated from *L. hyperborea* is a succinylated laminaran, which significantly enhanced the plant defense responses in tomato seedlings and induced cell wall modifications, suggesting a higher elicitor activity than the laminaran standard extracted from *L. digitata*. Thus, these data can contribute to the knowledge on this novel plant elicitor candidate and plant pathways identified. Moreover, this study also provides new information on the original laminaran composition of the macroalgae *L. hyperborea.*

## Figures and Tables

**Table 1:** ^13^C NMR chemical shifts (δ ppm) of LAM2 isolated from *Laminaria hyperborea*.

	COOH	C1	C3	C5	C2	C4	C6	-CH_2_-
3-linked Glc		102.53	84.15	75.58	73.25	68.08	60.66	
3,6-linked Glc			86.09					
6-succinyl Glc		102.53	84.15	74.36	70.79	69.21	63.17	
Succinate	175.69 179.46							43.86

**Table 2:** Gene expression analysis after treatments with LAM2 1%, LAM std 1% and water (Control). Genes are expressed by fold change with in bold and asterisk those significantly expressed (≤−1.5 or FC ≥ 1.5; *p*-value ≤ 0.05).

	LOX-D	PAL5	PPO-D	Pti-5	Worky28	Worky70-80
LAM2 1%/water	1.38	1.14	1.42	1.56 *	−1.68 *	−2.28 *
LAM std 1%/water	−1.00	1.16	−1.06	1.14	−2.32 *	−1.71 *
LAM2 1%/LAM std 1%	1.38	−1.02	1.50 *	1.36	−1.38	−1.33

## Data Availability

Authors declare no other data available from this study.

## Supplementary Materials

The following supporting information can be downloaded at: https://www.mdpi.com/article/10.3390/biom13101483/s1, Table S1: Monoclonal antibodies used in the immunocytochemical characterization of glycopolymers [34,35,36,37]; Supplementary Materials: ImageJ macro developed for fluorescence intensity measurements.
