# Supplementary material for: LAM2: An Unusual Laminaran Structure for a Novel Plant Elicitor Candidate
Source: Biomolecules. 2023 Oct 5;13(10):1483. doi: 10.3390/biom13101483 (PMC10605138; doi:10.3390/biom13101483)
Supplement: Supplementary file 1 [file biomolecules-13-01483-s001.zip › biomolecules-2642471-supplementary.pdf]

biomolecules

Supplemental data

LAM2: An Unusual Laminaran Structure for a Novel Plant Elicitor Candidate

Cathleen Mirande-Ney [1], Quentin Arnaudin [1], Gaëlle Durambur [1], Carole Plasson [1], Sophie Bernard [1,2], Christophe Chamot [2], Julie Grivotte [1], Narimane Mati-Baouche [1], Azeddine Driouich [1], Jeremy Brebion [3], Franck Hennequart [3], Patrice Lerouge [1] and Isabelle Boulogne [1,*]

[1] GLYCOMEV UR 4358, SFR Normandie Végétal FED 4277, Innovation Chimie Carnot, University of Rouen Normandie, IRIB, F-76000 Rouen, France

[2] Université de Rouen Normandie, Normandie University, INSERM, CNRS, HeRacLeS US 51 UAR 2026, PRIMACEN, F-76000 Rouen, France

[3] ALGAIA R&D Center, 91 Rue Edouard Branly, F-50000 Saint-Lô, France

* Correspondence: isabelle.boulogne@univ-rouen.fr

Table S1. Monoclonal antibodies used in immunocytochemical characterization of glycopolymers.

Glycopolymer	mAb	Epitope	References
Homogalacturonan	LM19	Homogalacturonan with low degree of esterification	Verhertbruggen et al., 2009 [34]
	LM20	Homogalacturonan with high degree of esterification	
Xyloglucan	LM15	Xylosylated xyloglucan (XXXG)	Marcus et al.,2008 [35]
Arabinogalactan protein	JIM13	βGlcA-(1,3)-αGalA-(1,2)-Rha	Knox et al., 1991 [36]<br>Yates et al., 1996 [37]

Supplementary materials: ImageJ macro developed for fluorescence intensity measurements

```
open();
path = getDirectory("image");
nomLong=getTitle();
nomCourt=File.getNameWithoutExtension(path+nomLong);
```

```
getDimensions(width, height, channels, slices, frames);
getPixelSize(unit, pixelWidth, pixelHeight);
if (slices>1) {
        run("Z Project...", "projection=[Average Intensity]");
}

run("Enhance Contrast", "saturated=0.35");
makeLine(460, 712, 3844, 2008);
waitForUser("Settings", "Please adjust the line to the region you need\nand be sure it
is wide enough.\n\nThen, press OK");

// Get profile and display values in "Results" window
run("Clear Results");
profile = getProfile();
for (i=0; i<profile.length; i++){
        setResult("Distance", i, i*pixelWidth);
        setResult("Value", i, profile[i]);
        updateResults;
}

saveAs("Results", path+nomCourt+".csv");


  // Plot profile

Plot.create("Profile", "X", "Value", profile);

run("Close All");
selectWindow("Results");
run("Close");
```
